# Alzheimer’s Disease-Related Protein Expression in the Retina of *Octodon degus*

**DOI:** 10.1371/journal.pone.0135499

**Published:** 2015-08-12

**Authors:** Lucia Y. Du, Lily Y-L. Chang, Alvaro O. Ardiles, Cheril Tapia-Rojas, Joaquin Araya, Nibaldo C. Inestrosa, Adrian G. Palacios, Monica L. Acosta

**Affiliations:** 1 School of Optometry and Vision Science, The University of Auckland, Auckland, New Zealand; 2 New Zealand National Eye Centre, The University of Auckland, Auckland, New Zealand; 3 Centro Interdisciplinario de Neurociencia de Valparaíso, Universidad de Valparaíso, Valparaíso, Chile; 4 Center for Aging and Regeneration (CARE), Department of Cell and Molecular Biology, Faculty of Biological Sciences, Pontificia Universidad Católica de Chile, Santiago, Chile; University of S. Florida College of Medicine, UNITED STATES

## Abstract

New studies show that the retina also undergoes pathological changes during the development of Alzheimer’s disease (AD). While transgenic mouse models used in these previous studies have offered insight into this phenomenon, they do not model human sporadic AD, which is the most common form. Recently, the *Octodon degus* has been established as a sporadic model of AD. Degus display age-related cognitive impairment associated with Aβ aggregates and phosphorylated tau in the brain. Our aim for this study was to examine the expression of AD-related proteins in young, adult and old degus retina using enzyme-linked or fluorescence immunohistochemistry and to quantify the expression using slot blot and western blot assays. Aβ4G8 and Aβ6E10 detected Aβ peptides in some of the young animals but the expression was higher in the adults. Aβ peptides were observed in the inner and outer segment of the photoreceptors, the nerve fiber layer (NFL) and ganglion cell layer (GCL). Expression was higher in the central retinal region than in the retinal periphery. Using an anti-oligomer antibody we detected Aβ oligomer expression in the young, adult and old retina. Immunohistochemical labeling showed small discrete labeling of oligomers in the GCL that did not resemble plaques. Congo red staining did not result in green birefringence in any of the animals analyzed except for one old (84 months) animal. We also investigated expression of tau and phosphorylated tau. Expression was seen at all ages studied and in adults it was more consistently observed in the NFL-GCL. Hyperphosphorylated tau detected with AT8 antibody was significantly higher in the adult retina and it was localized to the GCL. We confirm for the first time that Aβ peptides and phosphorylated tau are expressed in the retina of degus. This is consistent with the proposal that AD biomarkers are present in the eye.

## Introduction

In search of early biomarkers for Alzheimer’s disease (AD), attention has been directed to the retina, the lens and the visual pathway [1–4]. Several studies involving transgenic mouse models of familial AD observed specific pathological changes in the retina; some of which were similar to those found in human AD eyes [5,6]. However, these studies only explain the small number of familial cases. There are no major pathological differences in the brain between sporadic and familial cases in human [7] but there is inconclusive evidence about the pathological changes in the eye [8,9].

The disease course of AD is insidious and progressive with 95% of cases being sporadic [10]. Age is a major contributing factor and at advanced stages, AD pathology is commonly characterized by extracellular amyloid beta (Aβ) plaques and intracellular neurofibrillary tangles (NFT) in the cerebral cortex, along with cortical neurodegeneration [11]. The main constituent of amyloid plaques, Aβ protein, is known to be neurotoxic in its oligomeric non-fibrillar form, which can lead to mitochondrial dysfunction, oxidative stress and eventually cell death [12–14]. It has been postulated that Aβ protein could be the initiator of AD, leading to downstream pathological and degenerative changes that form the prevailing theory called the “amyloid hypothesis” [15]. Nevertheless, evidence also supports the neurotoxicity of tau proteins, which are the primary component of NFT [16].

The development of AD-related pathology in the retina could be the consequence of the expansion of neurodegeneration through the central nervous system, since the retina is a neural extension of the brain. However, there is also evidence to suggest that the molecular changes in the retina occur at a similar time as the brain, given that neurons and glial cells in the retina also have similar metabolic demands [17]. *In vivo* imaging of the retina of transgenic AD mice demonstrated a significant reduction in Aβ deposits when the animals were immunized against Aβ, which was in concomitant with reduced Aβ plaque burden in the brain [5]. This indicates that AD pathology in the retina is dynamic and reflects the progression of AD pathology in the brain.

There are very few animal models suitable for investigating whether the sporadic form of AD has associated pathological changes in the eye [18,19]. Inestrosa et al [20] and Ardiles et al. [21] have shown that *Octodon degus*, a rodent species endemic to South America, develop both Aβ and tau pathology in the brain, and cognitive decline as a result of aging [22]. The simplest explanation for the similarity of degus and human AD pathology is that the amino acid sequence of the Aβ proteins in degus shares 97.5% homology to the human Aβ [20,23]. In addition, the brain of degus is rich in acetylcholinesterase neurons, which are the main neurons affected in human AD as a response to Aβ deposits and hyperphosphorylated tau protein aggregates [24,25]. The severity of pathology appears to correlate with cognitive dysfunction, such as spatial and object recognition memory loss due to synaptic deregulation [21,26]. This is similar to the behavioral perturbations seen in human AD [27–29]. Here we have identified the expression of AD-related proteins in the retina of degus. We show that the expression is mainly localized to the nerve fiber layer and ganglion cell layer (NFL-GCL) and it is more significantly found in the retina of adult and old animals.

## Materials and Methods

### Animals

Degus were born and raised in the animal facility at the University of Valparaiso, Chile. The animals were housed under a 12:12 light/dark cycle in a controlled temperature environment (23±1°C) with water and food *ad libitum*. Retinae from 46 animals were collected for histological analysis and for protein collection. The animals were subjectively grouped into young, adult and old to allow the comparison of changes in protein expression. Twenty-six eyes were used for immunohistochemical detection of proteins in the young group (average 6.7 months; n = 9) adult group (average 28 months; n = 7) and old group (average 70 months; n = 10). Twenty one eyes were processed for western blot and slot blot procedures in the young group (average 5 months; n = 11) adult group (average 34 months; n = 5) and old group (average 76 months; n = 5). All experiments were approved by the University of Auckland Animal Ethics Committee (permit number: AEC 001138) and in accordance with the bioethics regulation of the Chilean Research Council (CONICYT) and Approved Animal Welfare Assurance (NIH A5823-01). All experiments were in accordance with the Association for Research in Vision and Ophthalmology statement for the use of animals in ophthalmic and vision research.

### Tissue fixation and sectioning

Degus were deeply anesthetized with halothane before decapitation. The eyes were collected post-mortem and fixed in 4% paraformaldehyde. Following fixation, the posterior cups were separated from the anterior eye structures, processed and sectioned using our standard protocols [30]. Vertical sections at 10 μm were obtained using a cryostat (CM3050, Leica Microsystems, Germany) and collected on glass slides (Superfrost Plus; Fisher, Pittsburgh, PA).

### Tissue staining and retinal measurements

The retinal sections were stained with toluidine blue [2% (w/v) Sigma Aldrich, Germany in H_2_O] for 30 seconds on a heat plate. The sections were mounted in glycerol, and protected with a coverslip for imaging. Staining was visualized using brightfield microscopy (40x objective). Total retinal thickness was calculated by measuring from the inner limiting membrane (ILM) to the external limiting membrane (ELM) using ImageJ (v1.34s. National Institute of Health, USA). The average retinal layer thickness was determined for 4 samples in the young and 4 samples in the adult group.

### Choice of antibodies and fluorescence immunohistochemistry

BLAST search on the NCBI website confirmed that the epitopes recognized by the antibodies used in this study (Table 1) are present with high homology in human and in degus. Antigen retrieval was conducted according to the antibody supplier’s instructions.

**Table 1:** Sequence alignment for the epitope of AD-related proteins in degus and human.

		Accession number	Epitope	Homology
**Aβ4G8**	Degu	XP_004627756.1	LVFFAEDV*	100%
	Human	NP_000475.1	LVFFAEDV	
**APPA4**	Degu	XP_004627756.1	KEGILQYCQEVYPELQ*	100%
	Human	NP_000475.1	KEGILQYCQEVYPELQ	
**Aβ6E10**	Degu	XP_004627756.1	DAEFRHDSGYEV**R**HQK	93.8%
	Human	NP_000475.1	DAEFRHDSGYEV**H**HQK	
**Tau5A6**	Degu	XP_004630049.1	------------ -T**LL**QDQ*	Sequence incomplete,
	Human	P10636.5	GLGDRKDQGGYT**MH**QDQ	partial alignment

*predicted sequences in NCBI sequence database

The primary antibodies (Table 2) were diluted in 3% NGS or 3% NDS in PBS and incubated overnight at room temperature. The negative control for the immunohistochemical procedure included omission of the primary antiserum, which was replaced by the antibody diluent followed by normal processing. The appropriate secondary antibodies were then applied for 3 hours at room temperature in the dark. The secondary antibodies used were goat anti-mouse or anti-rabbit IgG conjugated to Alexa Fluor 594 or 488 diluted to 1:500 using 3% NGS solution. Negative controls resulted in no labeling of the retina. The nuclear marker, 4',6-diamidino-2-phenylindole (DAPI, D9542, Sigma Aldrich), was also included in the secondary antibody solution at 0.125μg/ml. Quenching with copper sulphate [20% (w/v) Sigma] in water for an hour was a step in all immunohistochemical procedures before mounting the retina in the anti-fade medium, Citifluor.

**Table 2:** List of primary antibodies used for immunohistochemistry.

**Antigen**	**Antiserum**	**IsoType**	**Epitope**	**Dilution**	**Vendor**	**Reference**
**Glutamine synthetase**	Mouse anti-glutamine synthetase(monoclonal)	IgG2a	Amino acids 1–373 of sheep glutamine synthetase	1:500	BD Biosciences (610518)	
**Aβ4G8**	Mouse anti-Aβ (monoclonal)	IgG2b	Amino acids 17–24 of human Aβ	1:200	Covance (SIG-39220)	[5,52]
**A11**	Rabbit anti-Aβ oligomer A11(polyclonal)	IgG	Aβ oligomers that are independent of the amino acid sequence	1:20	Invitrogen(AHB0052)	[52]
**APPA4**	Mouse anti-APPA4 (monoclonal)	IgG1	Amino acids 66–81 at N terminus of APP	1:100	Millipore(MAB348)	[53]
**Aβ6E10**	Mouse anti-Aβ (monoclonal)	IgG1	Amino acids 1–16 of human Aβ	1:200	Covance (SIG-39320)	[5,52]
**Tau5A6**	Mouse anti-tau (monoclonal)	IgG1	Amino acids 19–46. Recognizes six isoforms from 45–58 kDa in human brain	1:100	Developmental Studies Hybridoma Bank (5A6)	[54]
**AT8**	Mouse anti-human PHF-Tau (monoclonal)	IgG1k	Phosphorylated Ser202/Thr205 in human PHF-tau	1:200	Thermo Scientific (MN1020)	[8]
**Tau S235**	Rabbit antihuman Tau phosphorylation site of Serine 235	IgG	Synthetic phosphopeptide Phosphorylated tau at Ser 235	1:1000	AbCam (ab30664)	
**β-actin**	Mouse anti- β- actin	IgG1	Recognizes the N-terminal end of the b-isoform of actin. (42 kDa)	1:4000	Sigma Aldrich (A1978)	

### Terminal deoxynucleotidyl transferase-mediated dUTP nick-end labeling staining (TUNEL) labeling

Cell death was investigated in young and adult retina using *In Situ* Cell Death Detection Kit (Roche Applied Science, Mannheim, Germany) as per the supplier’s instructions.

### Retina whole mount immunohistochemistry and quantification

Retinal pieces of 2 mm x 2 mm within the central (≤2 mm from optic nerve) and peripheral (≤2 mm from ora serrata) regions were cut and placed floating in 0.5% Triton-X100 with the innermost retinal layers facing up. The sections were permeabilized by freezing at -80°C before immunohistochemistry. The number of cells per unit area were counted in the GCL in 10,000 μm^2^ per retina (n = 3 animals) and plotted as cells/mm^2^.

### Indirect enzyme-linked immunohistochemistry

Horseradish peroxidase (HRP-DAB) staining was carried out using the EnvisionDual Link System-HRP (DAB+, Dako, Denmark) following the manufacture’s protocol. No counterstain was used in order to prevent masking of weak DAB staining. Phase contrast images were acquired using a Leica Microsystems DMRA2 microscope (Germany) with a Leica DC 500 camera under Ph3 40x objective lens. All retinal sections were photographed with the same exposure time of 215.4 ms.

### Congo red staining

The Benhold Method was used to stain tissues with Congo red [0.3% (w/v) Congo red, 1% (w/v) NaOH]. Sections were visualized using polarized light microscopy for detection of any green birefringent amyloid plaques. Images of Congo red staining were obtained under 40x objective lens.

### Western blot and slot blot

Retinal samples were homogenized in RIPA buffer supplemented with a protease inhibitor cocktail (Sigma-Aldrich P8340) and phosphatase inhibitors using a previously published method [21]. Retinal proteins (20 μg) were resolved by 10% SDS-PAGE and transferred to a PVDF membrane. This was followed by incubation with a primary antibody; then a secondary anti-goat peroxidase conjugated antibody (Pierce). Soluble Aβ oligomers were measured by slot-blot assays as described previously [21]. Band intensities in slot blots and western blots were visualized with ECL (Pierce) and scanned for densitometrical quantification using ImageJ software (National Institutes of Health). Total tau was assessed using anti-tau5A6 antibody (1:100). Tau phosphorylation was assessed with AT8 (1:1000) and anti-tau phospho S235 (1:1000) antibodies. Data are presented as relative values to the loading control protein.

### Confocal microscopy imaging

Retinal sections were visualized with Olympus FV1000 Confocal Microscope (Olympus, USA) at the Biomedical Imaging Research Unit of the University of Auckland using 60x and 100x oil immersion objective lenses. Whole mount retinae were imaged in the GCL.

### Image analysis

Images were converted into a 16-bit binary image using ImageJ software (National Institutes of Health). The number of pixels occupied by the antibody was measured in an area 200 μm by 25 μm corresponding to the NFL-GCL. The total area analyzed in five images was averaged and plotted as pixels/100 μm^2^. This method of semi-quantification only takes into account the area occupied by the marker regardless of the intensity of the signal.

### Statistical analysis

Data is displayed as mean ± SEM. Statistical analysis was performed using GraphPad Prism (GraphPad Software Inc, San Diego, CA). Unpaired, two-tailed Student’s *T*-tests were performed to determine significant differences in the expression of each AD-related marker between the young and adult retina or young and old retina. A *p* value of <0.05 was considered to be statistically significant.

## Results

### Retinal structure

**Fig 1:**
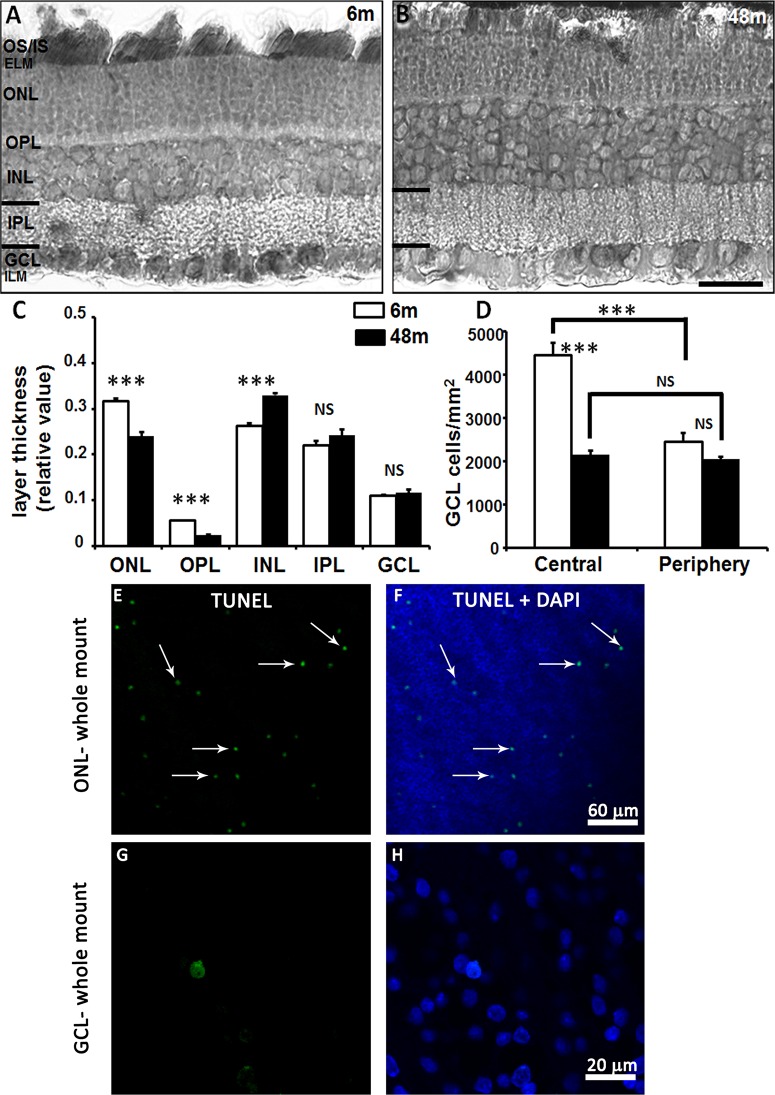
Light and confocal fluorescence micrographs illustrating the morphology of the retina in young and adult degus. Retinal sections from young and adult degus were stained with Toluidine blue (A, B). Quantification of retinal layer thickness (C) and ganglion cell density in young and adult degus (D). TUNEL labeling of the adult retina showed apoptotic cells in the ONL (E, F) but not in the GCL (G, H) in the retinal whole mount. Abbreviations: 6 months old (6m), 48 months old (48m), outer and inner segment of the photoreceptors (OS/IS), external limiting membrane (ELM), outer nuclear layer (ONL), outer plexiform layer (OPL), inner nuclear layer (INL), inner plexiform layer (IPL), ganglion cell layer (GCL), inner limiting membrane (ILM). Scale bar = 20 μm.

The histological appearance of the retina was examined to determine any group-related structural differences (Fig 1A and 1B). There were no overt changes in the arrangement and shape of the inner and outer segments (IS/OS) of the photoreceptor layer and the morphology of cell bodies in all the layers between the young and adult degus. The outer nuclear layer (ONL) and outer plexiform layer (OPL) of the central retina were significantly thicker in the young animals compared with the adults (Student’s t-test, p<0.0001; Fig 1C). Conversely, the inner nuclear layer (INL) was significantly thinner in the young degus (p<0.0001). The retinal GCL is composed of one layer of cells and to determine whether there were changes in the number of cells in this area we quantified GCL density. GCL density was significantly higher in the central retina compared to the periphery in young degus (45% more; Student’s t-test p<0.001), whereas in adults there were no statistically significant differences between the central and peripheral retina (p = 0.41; Fig 1D), as previously reported [31]). We observed a significant 52% decline in the number of cells per unit area in the adult central retina compared to the young (p<0.001). TUNEL positive cells were not observed in the young degus (n = 4) but they were observed in the adults in the ONL whole mount (Fig 1E and 1F). In the same retinal whole mount there were nearly no TUNEL positive cell in the GCL (n = 6; Fig 1G and 1H).

**Fig 2:**
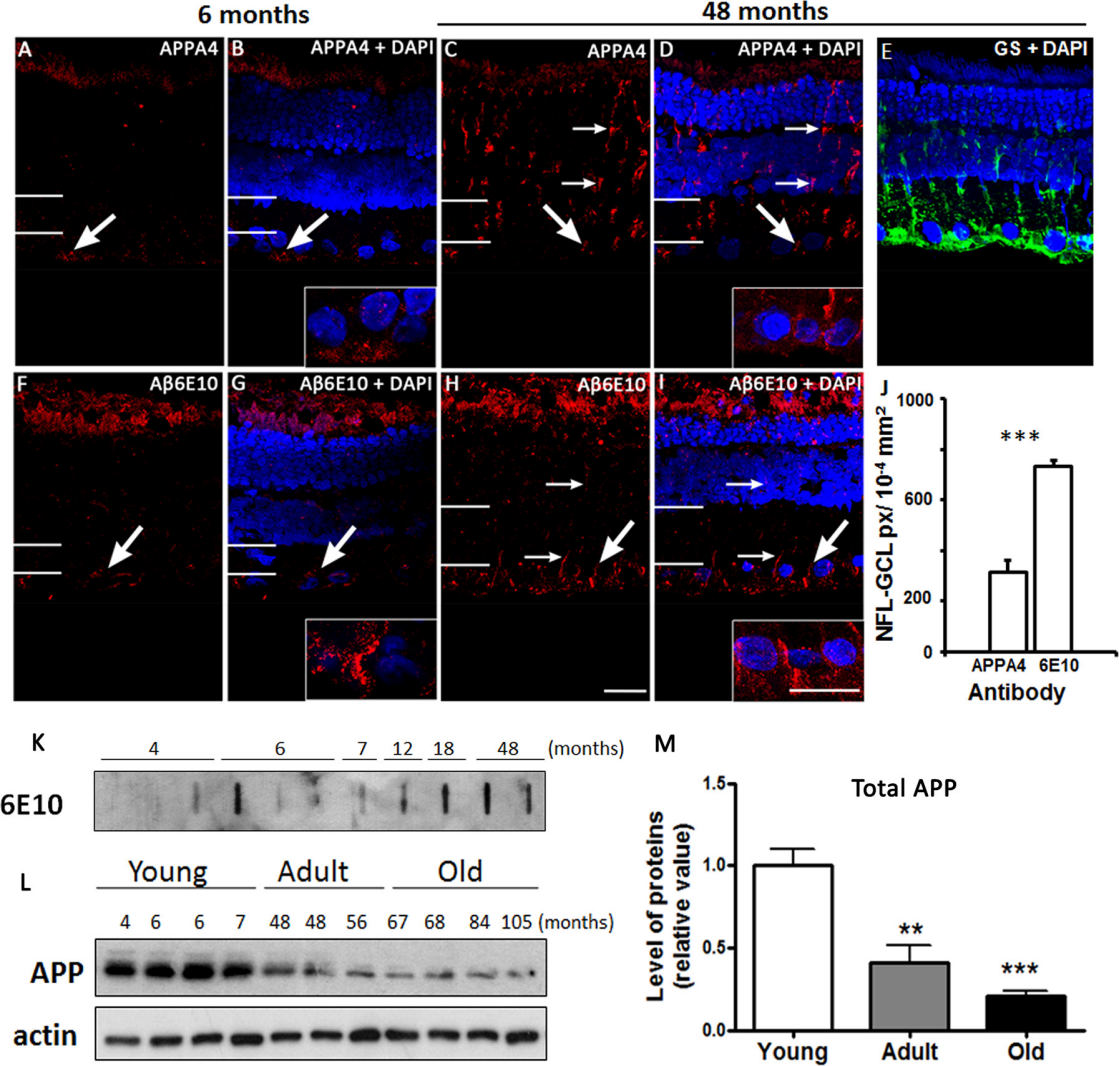
APP and Aβ peptide expression. Fluorescent immunolabeling of APP in 6 months old (A, B) and 48 months old (C, D) retinal sections. DAPI (blue) stains nucleus. GS labeling in adult retina (E). Aβ peptide expression in young (F, G) and adult (H, I) animals. The insert shows a magnification of the NFL-GCL area. Thin arrows indicate labeling in inner retina. Large arrows specify labeling in the GCL area. White lines on the left hand side of the image indicate the extent of the IPL. The number of pixels occupied by the antibody in the NFL-GCL is shown (J). Slot blot assay showing the level of Aβ protein detection with Aβ6E10 for young and adult degus (K). APP protein level was assayed using western blot (L). APP expression as a function of age was quantified in (M). Abbreviations: glutamine synthetase (GS), nerve fiber layer- ganglion cell layer (NFL-GCL), pixels (px). Scale bar = 20 μm.

Amyloid precursor protein (APP) and Aβ peptides are expressed in the degus retinaAPP expression was detected using APPA4 antibody, which marks the N-terminal part of the protein (Fig 2). In young degus, APP was clearly seen in the NFL. The fluorescence in the IS/OS was judged to be autofluorescence. APP expression was clearly seen in the adult retina from the NFL to the ELM. This linear-like pattern of expression is reminiscent of Müller cell labeling with glutamine synthetase (GS; Fig 2E). Aβ peptides marked with Aβ6E10 were found in the IS/OS and GCL in the young retina. Adult retina showed intense labeling in the IS/OS and throughout the retinal layers including the NFL. Compared with APP, Aβ6E10 labeling in the NFL-GCL was significantly higher in the adults (Student’s t-test, p< 0.001; Fig 2J). In order to understand how the expression levels of Aβ peptides and APP compare with age, we performed western blot and slot blot respectively (Fig 2K and 2L). Our results indicate that retinal APP expression is higher in young animals compared to the adults (Fig 2M). This was the opposite for Aβ peptides, given that expression in the young was not always seen but it was detected in most adult animals (Table 3).

**Table 3:** Number of animals employed in the molecular analysis.

	Animal code	Age (months)	Slot blot			Western Blot			
**Young**			**4G8**	**6E10**	**A11**	**Total tau**	**Ser235**	**AT8**	**APP**
	11	4	**-**	**-**	**-**	**+++**	**+**	**+**	**+++**
	27	4	**-**	**-**	**-**	**+++**	**+**	**+**	**+++**
	57	4	**-**	**+**	**-**	**+++**	**++**	**+**	**+++**
	383	4	**+**	**-**	**-**	**++**	**+**	**-**	**+++**
	45	6	**-**	**++**	**+**	**+++**	**++**	**+**	**++**
	10	6	**-**	**+**	**-**	**+++**	**++**	**+**	**++**
	315	6	**-**	**+**	**-**	**+++**	**+**	**+**	**+**
	63	6	**+**	**-**	**+**	**++**	**+**	**-**	**+++**
	64	6	**+**	**-**	**+**	**++**	**+**	**+**	**+++**
	30	7		**+**	**-**	**+++**	**+**	**+**	**++**
	47	7	**+**	**-**	**+**	**++**	**+**	**+**	**+++**
**Adult**	51	12		**++**		**+++**	**+++**	**+**	**+**
	313	18		**+++**		**+++**	**+++**	**+**	**++**
	694	48	**+**	**+++**	**+++**	**+++**	**+++**	**+++**	**+**
	680	48	**++**	**+++**	**+++**		**+++**	**+++**	
	681	48	**+++**		**+**	**++**	**++**	**++**	**++**
**Old**	655	56	**+++**		**++**	**++**	**++**	**+++**	**++**
	650	67	**+++**		**++**	**++**	**+++**	**+**	**+**
	269	68	**+++**		**++**	**++**	**+++**	**++**	**+**
	660	84	**+++**		**++**	**++**	**++**	**++**	**+**
	698	105	**+++**		**+++**	**++**	**+++**	**+++**	**+**

Values were qualified according respect to the loading control values as twice the value or more (+++), equal (++) or less than control but present (+). Absence of labelling in the Slot blot or WB is indicates with (-).

**Fig 3:**
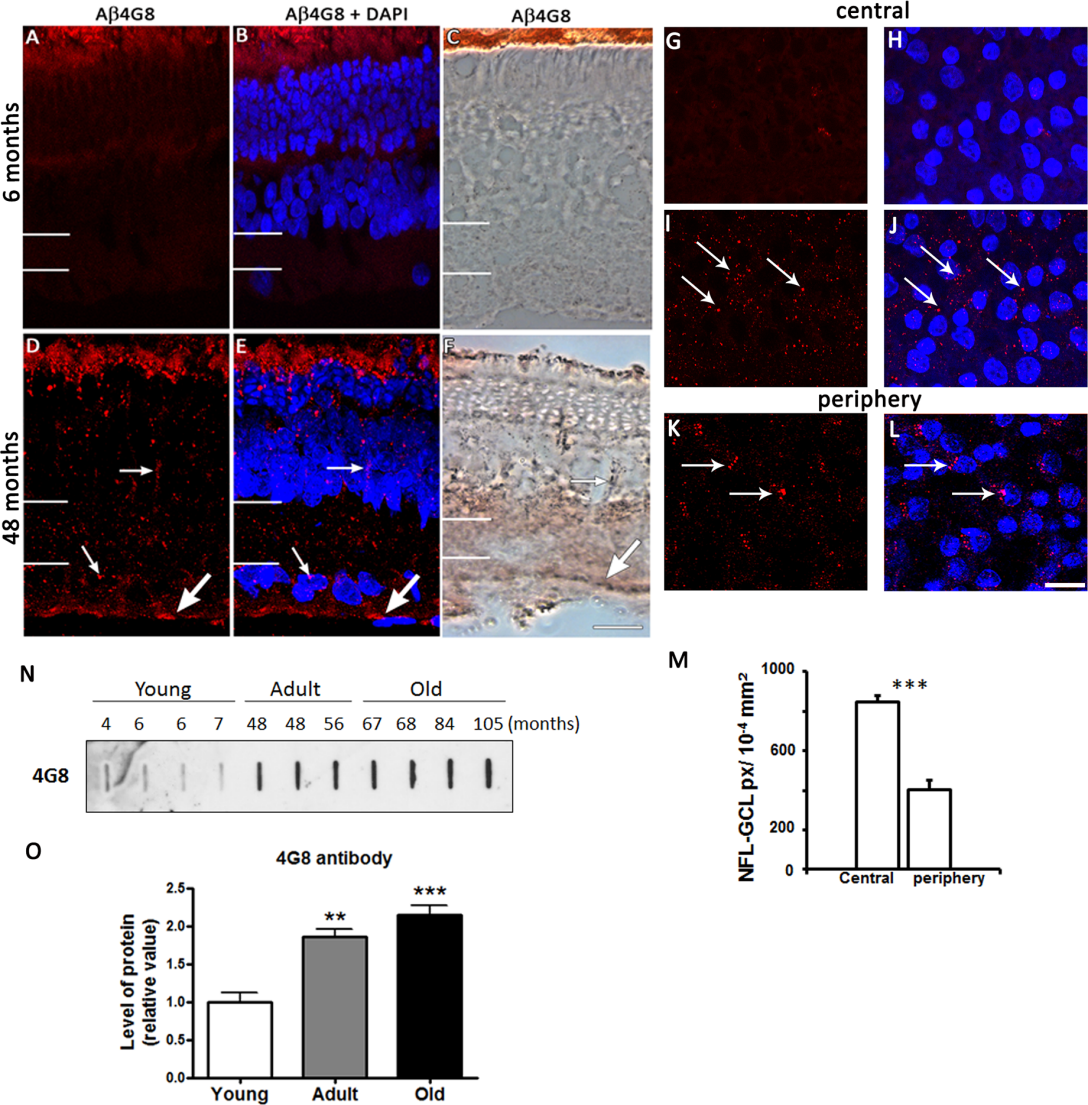
Expression of Aβ peptides. Fluorescent and enzyme-linked immunolabeling show Aβ4G8 labeling of Aβ peptides in 6 months old (A-C) and 48 months-old (D-F) retinal sections. DAPI (blue) stains nucleus. White lines on the left hand side of the image indicate the extent of the IPL. Aβ peptides were also labeled using fluorescence immunohistochemistry in the adult central (I, J) and peripheral (K, L) GCL. Arrows indicate the location of Aβ deposits. Negative control immunolabeling without the antibody is (G, H). The number of pixels occupied by the Aβ4G8 antibody in the central and peripheral NFL-GCL is shown (M). Slot blot assay for detection of Aβ peptides as a function of age (N) was quantified in (O). Abbreviations: nerve fiber layer- ganglion cell layer (NFL-GCL), pixels (px). Scale bar = 20 μm.

Immunohistochemistry labeling with another anti-Aβ peptide antibody, Aβ4G8, revealed a similar pattern to Aβ6E10 in young and adult retinae (Fig 3). No discernable fluorescent labeling was seen in the young degus retina except the normal residual autofluorescence of the retinal pigment epithelium (Fig 3A–3C). Expression was observed in all the retinal layers of the adult, particularly in the IS/OS and GCL. Aβ peptides in the GCL appeared as large aggregates compared to the smaller and fewer deposits seen in the rest of the retina (Fig 3D–3F). Both methods of immunohistochemistry using fluorescent- and enzyme-linked antibodies showed a similar pattern of Aβ peptide expression (Fig 3F). Omission of the antibody after the quenching procedure resulted in no labeling in the retina (Fig 3G and 3H). Re-labeling the same retina with Aβ4G8 resulted in specific labeling in the NFL and surrounding the GCL nuclei in both central and peripheral retina (Fig 3I–3L). Although it is not certain whether Aβ deposits are located extracellularly or intracellularly in ganglion cells (GCs) or in the synaptic ends of Müller cells, the labeling was specific to the antibody. Whole mount showed that Aβ protein were present mostly in the central retina (Fig 3I and 3J), which was confirmed by quantification of pixels occupied by the marker in the GCL (p<0.001; Fig 3M). The expression of proteins detected with the Aβ4G8 antibody was confirmed using slot blot (Fig 3N), and quantification of the expression indicated significantly higher expression in adult and older animals compared to the young (Fig 2O).

**Fig 4:**
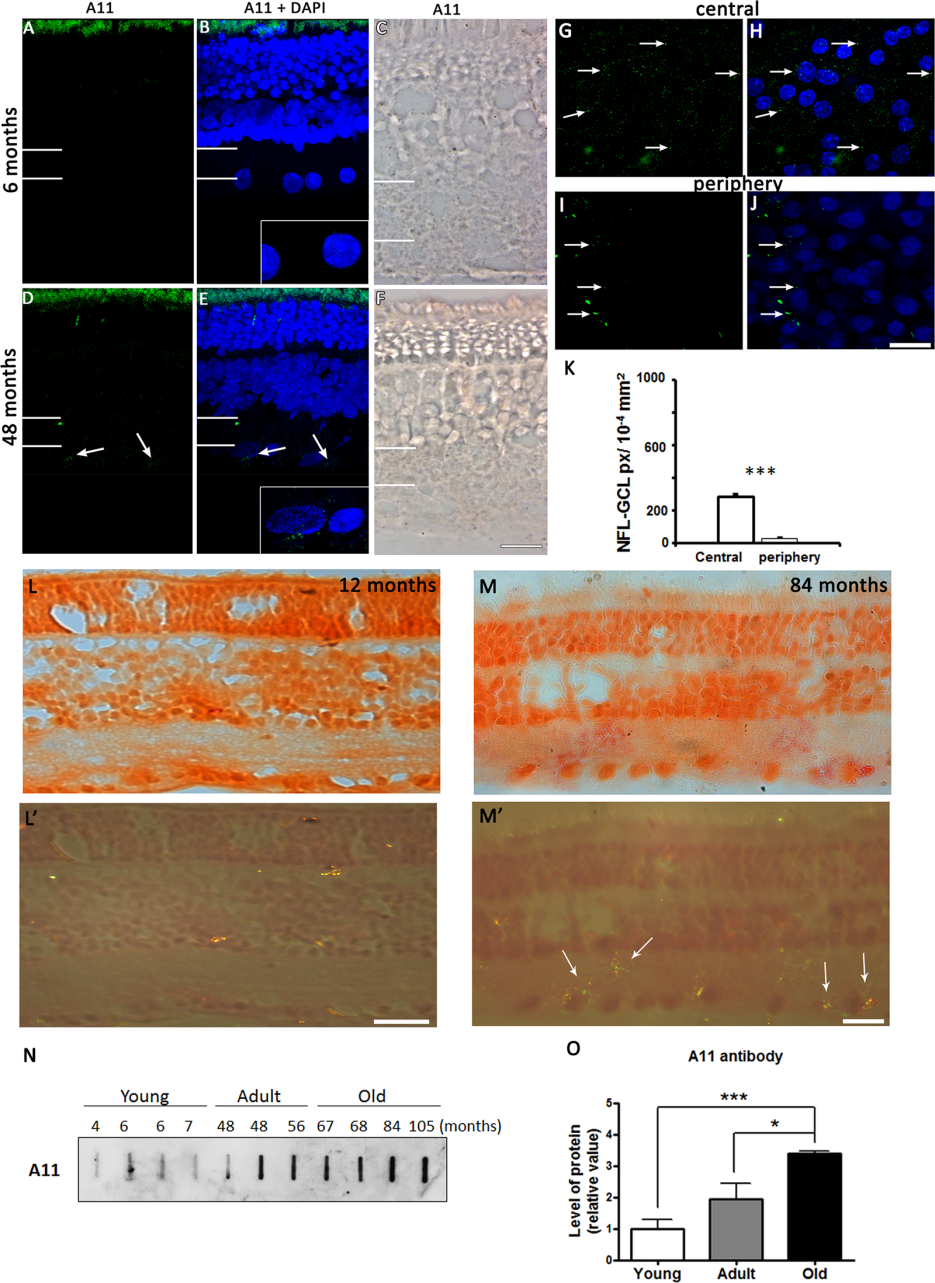
Expression of Aβ oligomers. Fluorescent and enzyme-linked immunolabeling show the absence of Aβ oligomers in 6 months old (A-C) and faint labeling in the adult, 48 months old (D-F) retinal sections. Aβ oligomers were also detected in 48 months old central (G, H) and peripheral (I, J) GCL. Arrows indicate the location of Aβ oligomers. The insert shows a magnification of the NFL-GCL. White lines on the left hand side of the image indicate the extent of the IPL. The number of pixels occupied by the A11 antibody in the central and peripheral NFL-GCL is shown (K). Congo red staining of a 12 month retina (L) and polarized light microscopy (L’) did not show presence of amyloid fibrils. Congo red was seen only in an 84 month old retina (M, M’). A11 detection of proteins in a slot blot as a function of development (N) shows the age-related expression of the peptide (O). Scale bar = 20 μm.

Aβ oligomers were detected in the retina. We observed no expression in the young retina in both retinal sections and whole mounts but they were detected in a slot blot (Fig 4A–4C). Adult retinal sections revealed weak labeling of Aβ oligomers (Fig 4D–4F). However, this expression was better observed in the whole mounts, which showed clear small punctuate labeling within the GCL (Fig 4G–4J). Similar to the distribution of Aβ peptides, there was a significantly higher level of Aβ oligomers in the central retina than in the retinal periphery (Fig 4K; Student’s t-test, p<0.01). We did not detect positive Congo red staining under polarized light in the young and adult retina (Fig 4L–4L’). Expression of Aβ peptides was seen in most of the old animals but Congo red staining was only clearly seen in the GCL and inner plexiform layer in a 84 month retina (Fig 4M) that was apple green under polarized light (Fig 4M’). We concluded that Congo red staining did not accurately reveal amyloid proteins in the retina. To verify whether detection of Aβ oligomers with A11 antibody was specific, we conducted a slot blot assay (Fig 4N) using the A11 antibody against soluble Aβ amyloid assemblies. The A11 antibody detected expression at all ages but the Aβ oligomers in was significantly higher in the old retina compared with the adults (Student’s t-test p<0.05) and with the young (Student’s t-test p<0.01; Fig 4O).

### Tau-related markers were present in the degus retina

**Fig 5:**
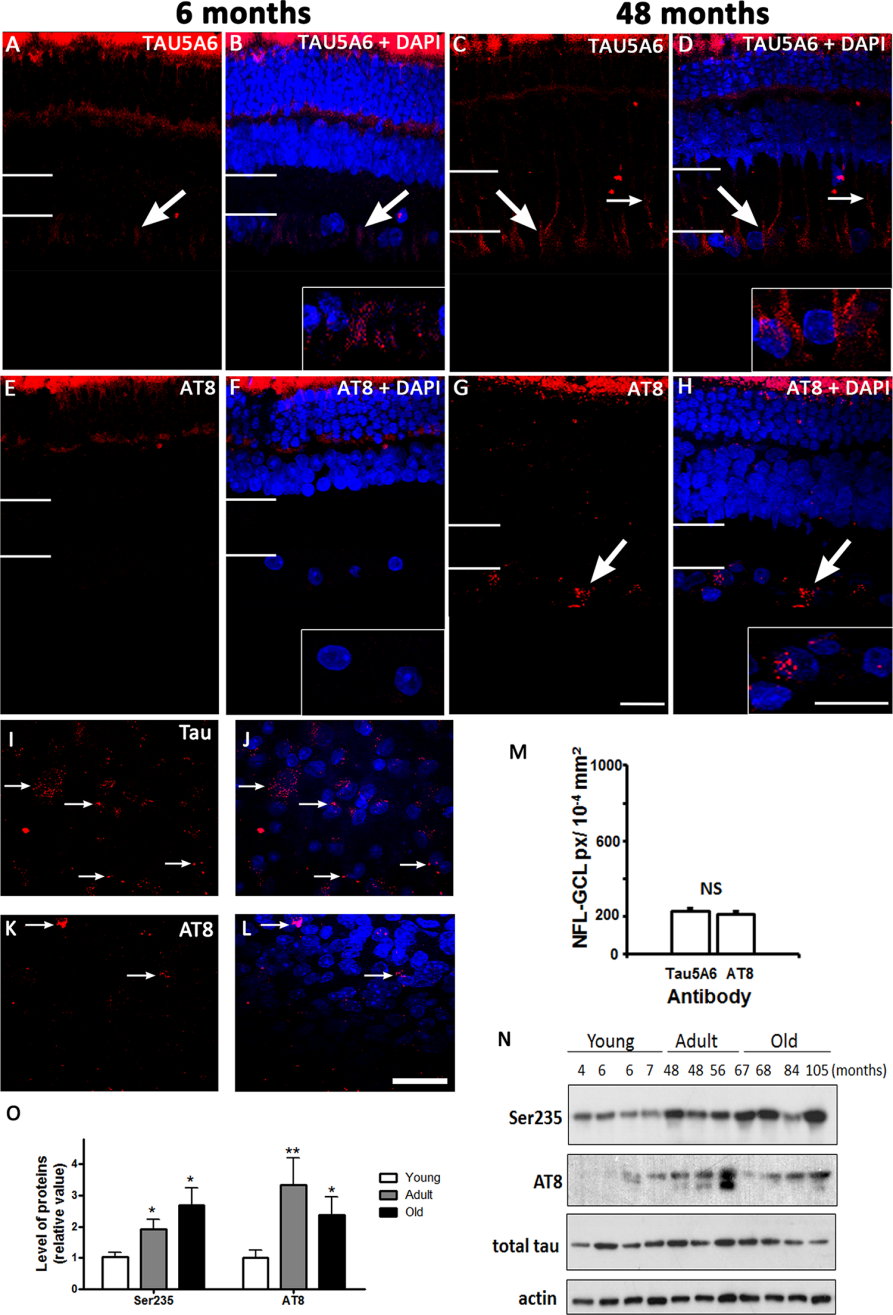
Tau and PHF-tau protein expression. Fluorescent immunolabeling shows tau expression in 6 months old (A, B) and 48 months old (C, D) retinal sections. DAPI (blue) stains nucleus. PHF-tau proteins labeled with AT8 in young (E, F) and adult (G, H) retinal sections. The insert shows a magnification of the NFL-GCL. Large arrows indicate positive labeling in the GCL area and thin arrows highlight the labeling pattern along the vertical axis, which is reminiscent of Müller cells. Thin white lines on the left hand side of the image indicate the extent of the IPL. Whole mount labeling using Tau5A6 (I, J) and AT8 in the adult central GCL (K, L). Arrows indicate the location of tau and phosphorylated tau respectively. The number of pixels occupied by the Tau5A6 and AT8 antibodies in the central NFL-GCL is shown (M). Western blot of phosphorylated tau and PHF levels in young and adult retinae using Ser235 and AT8 antibodies (N). Quantification of the bands shows an age-related increase in expression of phosphorylated tau and PHF detected with Ser235 and AT8 (O). Anti-actin was used as loading control. Scale bar = 20 μm

Another major pathological hallmark of AD is the presence of NFTs, which is comprised of paired helical filaments (PHF) made from hyperphosphorylated tau. Normal tau isoforms were weakly labeled in the GCL and OPL in the young animals compared to the adults (Fig 5A–5D). The tau expression pattern is similar to the labeling pattern of Müller cell end-feet in the adult retina (Fig 2E). Phosphorylated tau expression was not detected in the young degus with both fluorescent (Fig 5E and 5F) and enzyme-linked labeling (data not shown). However, western blot showed low expression in some young samples (Fig 5N and Table 3). This was the opposite for adults where fluorescent labeling was seen as small focal deposits in the GCL, surrounding the nuclei of the cells (Fig 5G and 5H). Whole mount of the GCL layer showed a similar pattern of labeling in this area for total tau and phosphorylated tau (Fig 5I–5L). We found no difference between the amount of tau and AT8 labeling in the NFL-GCL in the central retina of the adult animals (Fig 5M). Western blot results demonstrate that although the retinal level of total tau remains the same in young and adult degus, the level of phosphorylated tau detected using AT8 and Ser235 antibodies were higher in the adults (Fig 5N). The quantification showed the greatest amount was detected with Ser235and AT8 at 48 months and older (Fig 5O).

## Discussion

We have confirmed the presence of AD biomarkers in the retina of degus, which is a recently established natural model of AD. Aβ oligomers and phosphorylated tau were expressed predominantly in IS/OS of photoreceptors, GCL and NFL. Strong labeling was seen in the adult and old tissues but immunohistochemically there were no detectable NFT or Aβ plaques. Expression of Aβ in the adult was seen in the GCL but it was not associated with apoptotic cell death. The lower GCL density in the older animals could therefore be a reflection of GC loss through other mechanisms of cell death. On the other hand, positive TUNEL labeling was observed in the old degus ONL retina, which may be associated with the age-related apoptotic cell death and thinning of the ONL in the adult. The findings reinforce the concept that a cytotoxic effect of amyloid peptides may be present in the retina in the absence of Aβ plaque formation [32].

### Amyloid peptides and oligomers are expressed in the retina

Aβ aggregates in the brain are a common feature of AD [11], but in the retina little is known about its development and function. APP-KO mice do not show major visual acuity or functional retinal changes to suggest a major role of APP in basic visual function [33]. On the other hand, amyloidogenic cleavage of the transmembrane APP in the retina has been shown to be associated with increased GC loss [34]. In degus, the higher expression of Aβ peptides labeled with Aβ4G8 in the GCL is consistent with the amyloidogenic process occurring in degus retina. This is consistent with the observations in AD human patients and transgenic AD mice [5,35]. However, Aβ peptides appear at a much lower level in the retina compared to the brain [21], perhaps due to the low expression level of β-secretase in the retina [36]. The small and discrete Aβ oligomer labeling observed in the central GCL in adult degus does not resemble the Aβ plaque-like deposits in the AD human brain and in the retina and brain of transgenic mice [5]. The oligomeric form of Aβ peptides was not as extensively distributed in the degus retina and was seen to be mainly concentrated in the central region. This spatial localization of Aβ oligomers mirrors the expression pattern of Aβ peptides indicating that the retina contains components necessary for the formation of these oligomers, akin to the brain [21].

### Phosphorylated tau in the degus retina

Phosphorylated tau was seen in degus GCL and NFL, which is consistent with the findings of PHF expression in retinal tissues from AD donors, P301S tau mice and Tg2576 transgenic mice overexpressing tau and APP with the Swedish mutation [37]. In degus, there was a trend suggestive of a disease- and age-related pattern of phosphorylated tau expression when detected by tau ser235 and AT8 antibodies. Interestingly, these tau residues are known to be phosphorylated at different times. For instance, phosphorylated ser235 has been identified in normal and AD brains [38], and has been associated with pre-NFT stages [39], whilst AT8 has been related to abnormally phosphorylated tau in PHF and NFT in AD brains [40,41]. Pre-NFT stages were seen in the adult brain, confirming that a reason for only seeing expression in adult and older degus is the age-related expression of tau pathology. However, old degus retina did not show NFT, allowing us to conclude that NFT do not form in the retina.

### Implications of amyloid peptides and tau pathology in the retina

The spatial localization of Aβ peptides and phosphorylated tau may have functional and pathological implications in the retina. Expression of Aβ peptides and phosphorylated tau was significant in aging animals and was more prominent in the central than in the peripheral retina. While vision loss in human AD has been associated with central vision problems [42], we consider that a direct functional comparison with the expression of peptides in central retina in degus cannot be made at this stage until visual acuity data is obtained. We can only suggest that the high expression of these peptides in the central retina may be related to the arrangement GCs as an area centralis [31]. In vertebrates that possess area centralis, glial and displaced amacrine cells in the GCL are more evenly distributed so that any changes in the central retina are likely due to the GCs [43]. Nevertheless, the expression seen in degus retina suggest that non-tangle-forming PHF may have deleterious downstream effects on the functionality and survivability of GCs. Based on the recently revised tau hypothesis on AD, hyperphosphorylated tau aggregates can act as a toxin to enhance the neuronal degeneration caused by the toxicity of Aβ oligomers and the accompanying oxidative and inflammatory responses. The preferential deposition of PHF and Aβ oligomers in the adult retina, specifically in the GCL suggests that GC loss in aged animals may be related to the protein expression. However, Aβ oligomers have been proposed to cause GC death in other retinal diseases such as glaucoma [44]. Furthermore, inhibitors of β-secretase demonstrate protective effects against GC death [45]. GC degeneration in AD retina is slightly more pronounced in the periphery [46–48]. Given that regional expression of Aβ-related proteins was not associated with TUNEL labeling of GCs, further investigation is required to determine what mechanisms cause the progressive degeneration of GCs in degus.

### Degus as a model in comparison with transgenic animals

Degus is a novel animal model for AD [20–22,49]. A major advantage of the degus over traditional AD transgenic animal models is that they are not genetically modified and naturally develop AD under the influence of environmental factors and systemic health. Therefore, degus represent the sporadic form of AD, which contributes to 95% of all human AD cases. Although genetics does seem to be one of the contributing factors of sporadic AD [50], its significance is not as clear as in familial AD in which mutations in three genes (APP, presenilin-1, PS1 and presenilin-2, PS2) have been identified to be accountable. Degus are also a model for type II diabetes, which supports the idea that vascular inefficiency plays a role in the etiology of sporadic microvascular AD [51]. In addition, the social behaviors of degus are more representative of the complex social human behavior compared to other lab rodents [26]. However, one of the disadvantages of the degus model is their long life span (9–10 years in standard lab conditions), and heterogeneity in AD prevalence in aged animals. This makes age-related studies costly and data analysis needs to be done case by case to accommodate for variability, as not all animals are affected. Nevertheless, this same argument reinforces the value of degus as a natural model in which to study mechanisms of neurodegeneration that involve pathways other than apoptotic cell death.

## Conclusion

Amyloid peptides, oligomers and phosphorylated tau were detected with a higher incidence in the retina of adult animals, which suggest that degus is a promising model for studying AD biomarkers in the eyes. The pattern and sparse expression of Aβ oligomers and phosphorylated tau indicate that the retina metabolism and aging process is similar to the brain. However, the absence of tangles and plaques suggest the model is different from the human condition. Therefore, degus provide the opportunity to better understand the etiology and pathogenesis of the disease using the eye as a model of amyloidogenic and tau processing. Additionally, the accessibility of the retina and the plethora of imaging technologies for *in vivo* analysis of the eye allow for the exploration of common factors presented in the brain and ocular tissues and the assessment of causal theories implicated in AD.
